# The Gastroprotective Effect of Naringenin against Ethanol-Induced Gastric Ulcers in Mice through Inhibiting Oxidative and Inflammatory Responses

**DOI:** 10.3390/ijms222111985

**Published:** 2021-11-05

**Authors:** Wei-Sung Li, Shih-Chao Lin, Chien-Hui Chu, Yu-Kang Chang, Xiang Zhang, Chi-Chien Lin, Yu-Tang Tung

**Affiliations:** 1 Department of Chinese Pharmaceutical Sciences and Chinese Medicine Resources, College of Chinese Medicines, China Medical University, Taichung 404, Taiwan; weisungli@tari.gov.tw; 2 Plant Pathology Division, Taiwan Agricultural Research Institute, Council of Agriculture, Executive Yuan, Wufeng 413, Taiwan; 3 Bachelor Degree Program in Marine Biotechnology, College of Life Sciences, National Taiwan Ocean University, Keelung 202, Taiwan; sclin@mail.ntou.edu.tw; 4 Department of Medical Laboratory, Taichung Armed-Forces General Hospital, Taichung 406, Taiwan; chien-hui@803.org.tw; 5 The iEGG and Animal Biotechnology Center, Institute of Biomedical Science, National Chung-Hsing University, Taichung 402, Taiwan; 6 Department of Medical Research, Tungs’ Taichung Metro Harbor Hospital, Taichung 433, Taiwan; t12193@ms.sltung.com.tw; 7 Department of Nursing, Jen-Teh Junior College of Medicine and Management, Miaoli 356, Taiwan; 8 Department of Molecular Medicine and Surgery, Karolinska Institute, SE-17177 Stockholm, Sweden; xiang.zhang@ki.se; 9 Department of Medical Research, China Medical University Hospital, Taichung 404, Taiwan; 10 Department of Medical Research, Taichung Veterans General Hospital, Taichung 407, Taiwan; 11 Department of Pharmacology, College of Medicine, Kaohsiung Medical University, Kaohsiung 807, Taiwan; 12 Department of Biotechnology, Asia University, Taichung 413, Taiwan; 13 Graduate Institute of Biotechnology, National Chung Hsing University, Taichung 402, Taiwan; 14 Cell Physiology and Molecular Image Research Center, Wan Fang Hospital, Taipei Medical University, Taipei 116, Taiwan

**Keywords:** gastric ulcers, naringenin, ethanol, inflammation, oxidative stress

## Abstract

Naringenin is a major flavanone found in grapes, tangelos, blood oranges, lemons, pummelo, and tangerines. It is known to have anti-inflammatory, antioxidant, anticancer, antimutagenic, antifibrogenic, and antiatherogenic pharmacological properties. This study aims to investigate the anti-inflammatory effects of naringenin in ethanol-induced gastric damage in vivo and ethanol-stimulated KATO III cells in vitro. Our results showed that pretreatment with naringenin significantly protected mice from ethanol-induced hemorrhagic damage, epithelial cell loss, and edema with leucocytes. It reduced gastric ulcers (GU) by suppressing ethanol-induced nuclear factor-κB (NF-κB) activity and decreasing the levels of nitric oxide (NO), malondialdehyde (MDA), tumor necrosis factor-α (TNF-α), interleukin-6 (IL-6), interleukin-8 (IL-8), and myeloperoxidase (MPO). In addition, pretreatment with naringenin might inhibit the secretion of TNF-α, IL-6, and IL-8, as well as the proteins cyclooxygenase-2 (COX-2) and inducible nitric oxide synthase (iNOS) via the suppression of NF-κB and mitogen-activated protein kinase (MAPK) signaling in ethanol-stimulated stomach epithelial KATO III cells. Together, the results of this study highlight the gastroprotective effect of naringenin in GU of mice by inhibiting gastric secretion and acidity, reducing inflammation and oxidative stress, suppressing NF-κB activity, and restoring the histological architecture. These findings suggested that naringenin has therapeutic potential in the alleviation of ethanol-induced GU.

## 1. Introduction

Gastric ulcer (GU) is one of the most common gastric diseases [1,2]. GU is a complex multi-factor process caused by the imbalance between aggressive and protective factors in the gastric mucosa [2]. The reasons for onset include the presence of *Helicobacter pylori*, decreased blood flow, increased gastric acid secretion and pepsin activity, imbalanced bile salt secretion, and decreased mucus and bicarbonate secretion [3,4]. In addition, acute GU often occurs due to alcohol consumption, non-steroidal anti-inflammatory drugs (NSAID) ingestion, and physiological, psychological or both stress [1,5], which can also lead to severe upper gastrointestinal bleeding with high mortality and morbidity [6]. Ethanol is a well-known gastric-mucosal-damaging agent [7,8]. Excessive intake of ethanol is one of the main causative agents of GU. Ethanol can also cause acute gastric mucosal damage [7,8]. Ethanol exposure rapidly activates neutrophils, which leads to oxidative stress by increasing the production of reactive oxygen species (ROS) and pro-inflammatory cytokines, which results in damage to the gastric mucosa [9]. Previous studies demonstrated that pro-inflammatory cytokines and oxidative homeostasis, e.g., nitric oxide (NO), malondialdehyde (MDA), glutathione (GSH), and superoxide dismutase (SOD), play an important role in the regulation of ethanol-induced acute GU [7,10,11,12]. Therefore, the ethanol-induced GU animal model is often used to evaluate anti-ulcer activity of natural products and drugs.

Several drugs (such as proton pump inhibitors, M1-receptor blockers, and H2-receptor antagonists) have been used in the treatment of GU, but the continuous and prolonged use of these drugs may result in serious adverse effects, including erectile dysfunction, arrhythmia, gynecomastia, and hematopoietic changes [13]. Therefore, recently, individuals have become increasingly interested in finding more effective and/or new biologically active compounds for the treatment of GU from various natural products [14,15,16,17]. Since GU is a multi-causal disease, new anti-GU compounds should have several properties, such as the ability to reduce gastric secretion and inflammation, as well as to enhance the endogenous defensive capacity of the gastric mucosa [1].

Naringenin is a major flavanone found in grapes, tangelos, blood oranges, lemons, pummelos, and tangerines [18]. The concentration of naringenin in grapefruit juice is estimated to be 1283 μmol/L (349 mg/L) [19]. In previous studies, it was found that naringenin exhibits anti-inflammatory [20,21], liver protection [22], antioxidant [23], anticancer [24], antimutagenic [25], antifibrogenic [26] and antiatherogenic [27] properties. Motilva et al. [28] pointed out that naringenin exerts protective mucosal activity in ethanol-induced gastric lesions in rat. However, at this juncture, we have no definite information about the protective mechanism of naringenin against ethanol-induced GU. To explore the gastroprotective mechanism of naringenin on against ethanol-induced GU. Therefore, this study aims to investigate the gastroprotective effect of naringenin against ethanol-induced GU in the cellular and animal models.

## 2. Results

### 2.1. Effects of Naringenin on Ethanol-Induced Acute Gastric Injury

**Figure 1:**
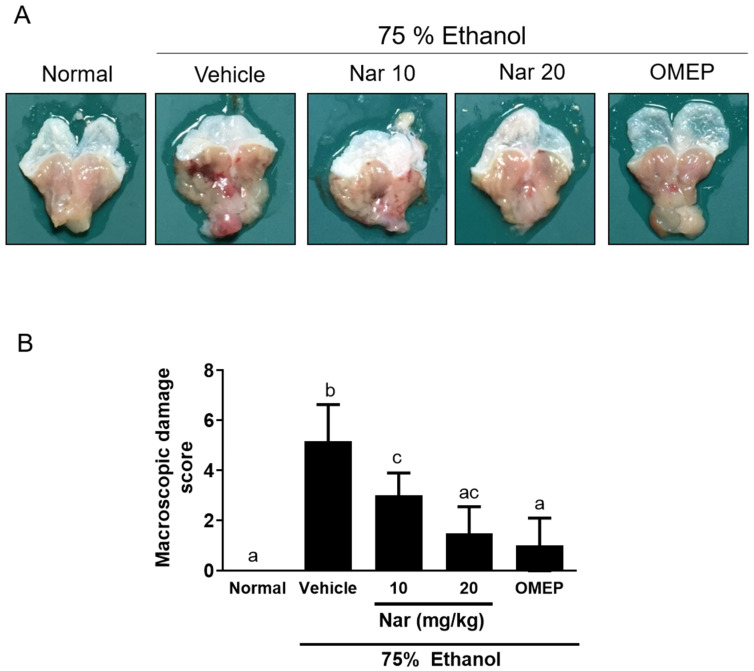
Naringenin improved the macroscopic morphology of gastric damage induced by ethanol in mice. C57BL/6 mice were randomly assigned to 5 different groups (6 mice per group). The vehicle (10% dimethyl sulfoxide (DMSO) and 90% glyceryl trioctanoate), naringenin (Nar, 10 and 20 mg/kg) and omeprazole (OMEP, 20 mg/kg; used as a positive control) were given orally for a period of 3 days. After fasting for 12 h prior to the experiment, mice were fed orally with ethanol (0.5 mL/100 g body weight) to induce the acute ulcer. After 4 h, mice were sacrificed. In the normal control group, the mice only received vehicle (Normal). (**A**) Representative photos of the dissected stomach. (**B**) The scores for macroscopic gastric damage. The data obtained from individual animal samples per group were averaged (*n* = 6); values represent mean ± standard deviation (SD). Statistical comparison was analyzed by a one-way ANOVA followed by Tukey’s multiple comparison tests. Bars not sharing a common letter represent a statistically significant difference (*p* < 0.05).

**Figure 2:**
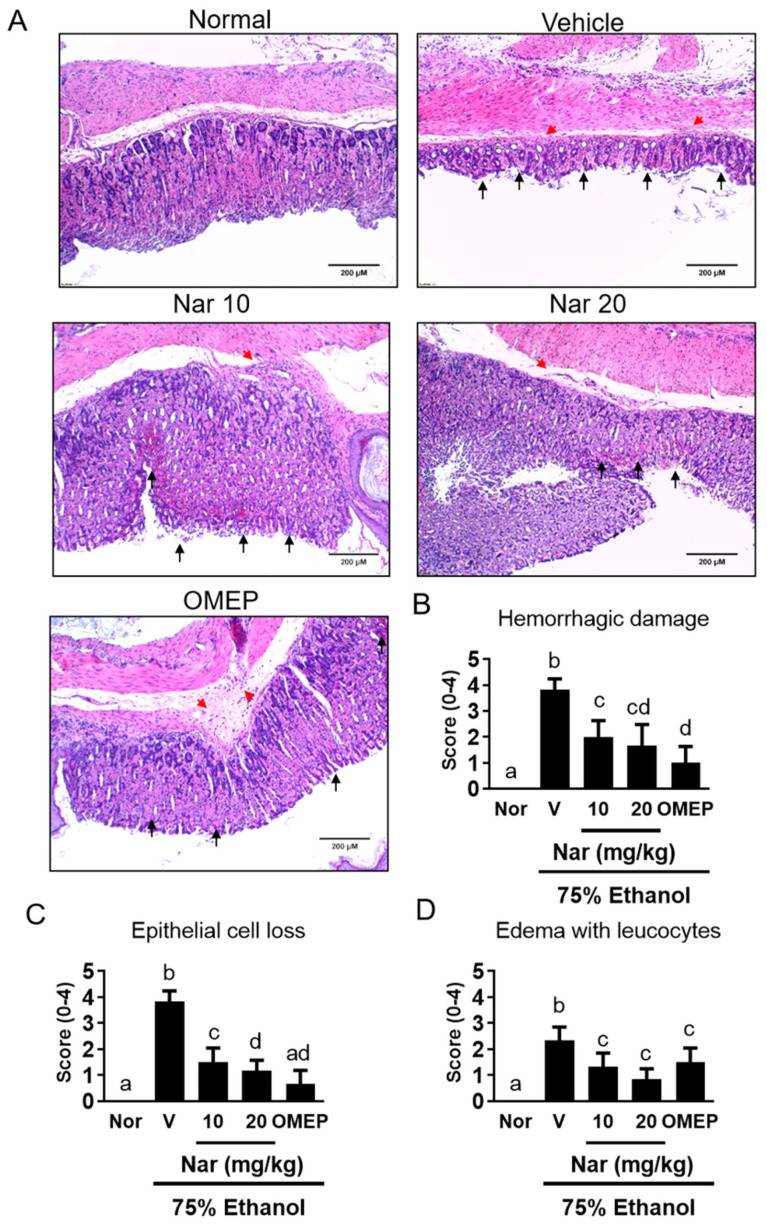
(**A**) Histological assessment of the effects of naringenin on acute gastric mucosal injuries in ethanol-induced mice. The gastric tissue (*n* = 6/group) was fixed with 4% paraformaldehyde and sectioned for HE staining (magnification ×100). Red arrowheads indicated the presence of edema, hemorrhagic damage. Black arrowheads indicated the loss of epithelial cells.. The scores for (**B**) hemorrhagic damage, (**C**) epithelial cell loss and, (**D**) edema with leucocytes. The vehicle (10% dimethyl sulfoxide (DMSO) and 90% glyceryl trioctanoate), naringenin (Nar, 10 and 20 mg/kg) and omeprazole (OMEP, 20 mg/kg; used as a positive control) were given orally for a period of 3 days. After fasting for 12 h prior to the experiment, mice were fed orally with ethanol (0.5 mL/100 g body weight) to induce the acute ulcer. After 4 h, mice were sacrificed. The data obtained from individual animal samples per group were averaged (*n* = 6); values represent mean ± standard deviation (SD). Statistical comparison was analyzed by a one-way ANOVA, followed by Tukey’s multiple comparison tests. Bars not sharing a common letter represent a statistically significant difference from each other (*p* < 0.05). In the normal control group, the mice only received vehicle (Normal). Nor, normal; V, vehicle.

To examine the gastroprotective effect of naringenin in vivo, adult Balb/c mice were pretreated with naringenin (10 or 20 mg/kg) for 3 days prior to the induction of acute GU by orally feeding 75% ethanol. Omeprazole is a proton pump inhibitor and is widely used in diseases caused by hyperacidity, such as GU [29]. Therefore, omeprazole is the positive control in the study. After ethanol induction, mice were sacrificed. Macroscopic and histological damage of the gastric mucosa is shown in Figure 1 and Figure 2, respectively. Oral administration of ethanol induced a large area of hemorrhagic ulcerative gastric lesions (Figure 1A). The severity of the damage was significantly decreased in mice pretreated with naringenin (10 and 20 mg/kg), as well as in mice pretreated with omeprazole (20 mg/kg) (Figure 1B). Furthermore, ethanol administration caused a significant alteration in the gastric epithelium, including hemorrhagic damage, epithelial cell loss, and edema with leucocytes (Figure 2). However, pretreatment with naringenin and omeprazole significantly alleviated ethanol-induced epithelial damage and contributed to preservation of the structure of the gastric wall (Figure 2). These macroscopic and histological examinations indicated that naringenin ameliorated ethanol-induced gastric mucosal damage.

### 2.2. Effect of Naringenin on the pH Value and the Content of Mucus in the Stomach

**Figure 3:**
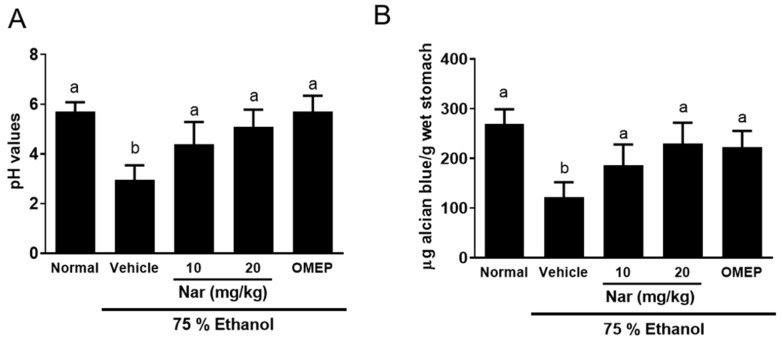
Effects of naringenin on (**A**) the pH value and (**B**) the content of mucus in the stomach. The naringenin (Nar, 10 and 20 mg/kg) and omeprazole (OMEP, 20 mg/kg; used as a positive control) were given orally for a period of 3 days. After fasting for 12 h prior to the experiment, mice were fed orally with ethanol (0.5 mL/100 g body weight) to induce the acute ulcer. After 4 h, mice were sacrificed. The data obtained from individual animal samples per group were averaged (*n* = 6); values represent mean ± standard deviation (SD). Statistical comparison was analyzed by a one-way ANOVA followed by Tukey’s multiple comparison tests. Bars not sharing a common letter represent a statistically significant difference from each other (*p* < 0.05).

Alcian blue is an indicator of the mucus content in gastric mucosa. Mice with gastric lesions showed a significant reduction in pH value (Figure 3A) and Alcian blue binding capacity of gastric mucosa (Figure 3B) compared to the normal control group. However, pretreatment with naringenin (10 or 20 mg/kg) and omeprazole (20 mg/kg) could significantly increase the pH value (Figure 3A) and the Alcian blue binding capacity of the gastric mucosa (Figure 3B) in gastric lesions of mice compared to those of the control group. These results revealed that naringenin ameliorated ethanol-induced elevation in gastric pH value and mucosal content.

### 2.3. Effect of Naringenin on Ethanol-Induced Oxidative Stress

**Figure 4:**
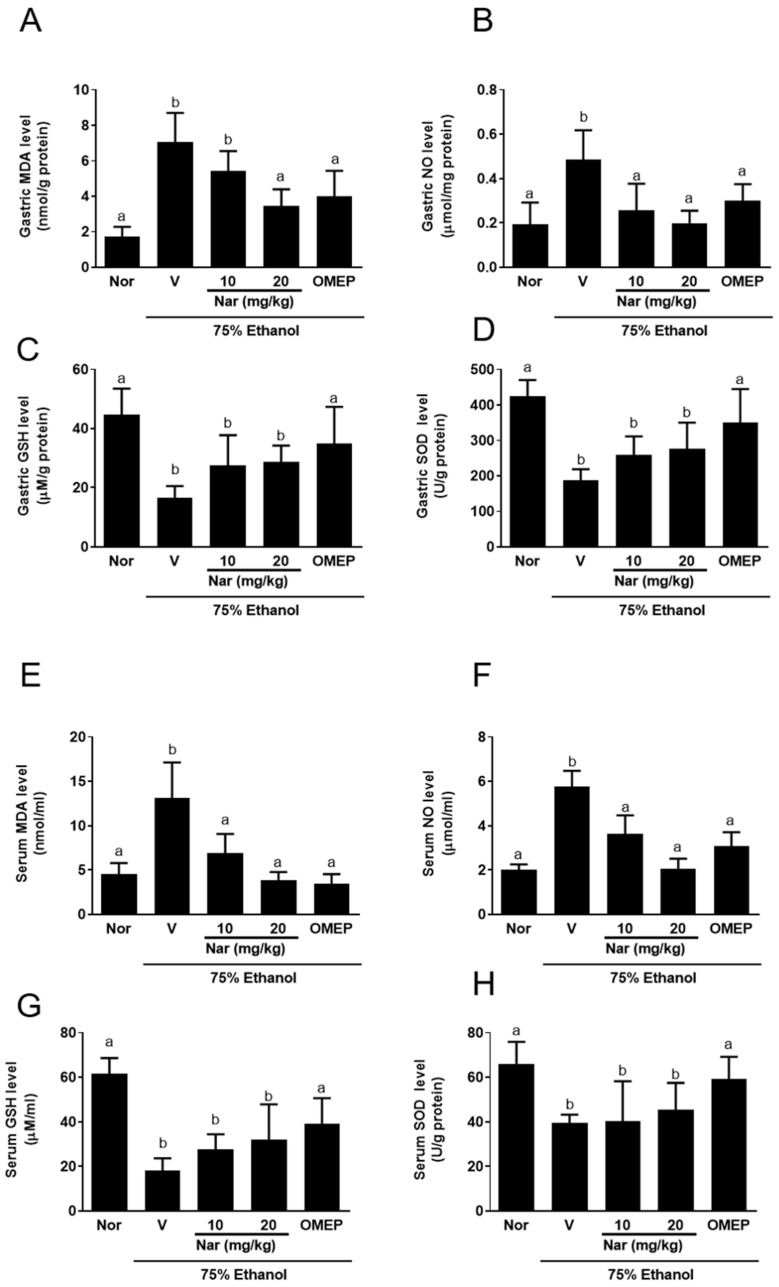
Effect of naringenin on ethanol-induced oxidative stress. The levels of nitric oxide (NO), malondialdehyde (MDA) and, GSH and SOD in the stomach homogenate (**A**–**D**) and the serum (**E**–**H**) were elevated by ulcer induction and lowered by pretreatment with the naringenin. The vehicle (10% dimethyl sulfoxide (DMSO) and 90% glyceryl trioctanoate), naringenin (Nar, 10 and 20 mg/kg) and omeprazole (OMEP, 20 mg/kg; used as a positive control) were given orally for a period of 3 days. After fasting for 12 h prior to the experiment, mice were fed orally with ethanol (0.5 mL/100 g body weight) to induce the acute ulcer. After 4 h, mice were sacrificed. The data obtained from individual animal samples per group were averaged (*n* = 6); values represent mean ± standard deviation (SD). Statistical comparison was analyzed by a one-way ANOVA followed by Tukey’s multiple comparison tests. Bars not sharing a common letter represent a statistically significant difference from each other (*p* < 0.05). In the normal control group, the mice only received vehicle (Normal). Nor, normal; V, vehicle.

Ethanol-induced gastric damage is usually accompanied by oxidative stress in gastric tissue [7]. Therefore, we examined the levels of oxidative stress biomarkers including NO, MDA, GSH, and SOD in gastric homogenates (Figure 4A–D) and serum (Figure 4E–H). The gastric and serum NO (Figure 4A,E) and MDA (Figure 4B,F) concentrations were significantly elevated by ethanol administration, while pretreatment with naringenin and omeprazole significantly reduced the elevation of NO and MDA in the gastric tissue and serum. In addition, naringenin recovered the activities of the antioxidant enzyme GSH (Figure 4C,G) and SOD (Figure 4D,H) in both gastric tissues and serum that were decreased by ethanol administration. Unfortunately, this recovery of activity was not deemed statistically significant. The above results suggested that naringenin has a protective effect on gastric mucosa, caused a reduction in NO and MDA levels, and slightly enhanced GSH and SOD activity in gastric tissue and serum.

### 2.4. Effect of Naringenin on Ethanol-Induced the Production of Inflammatory Factors

**Figure 5:**
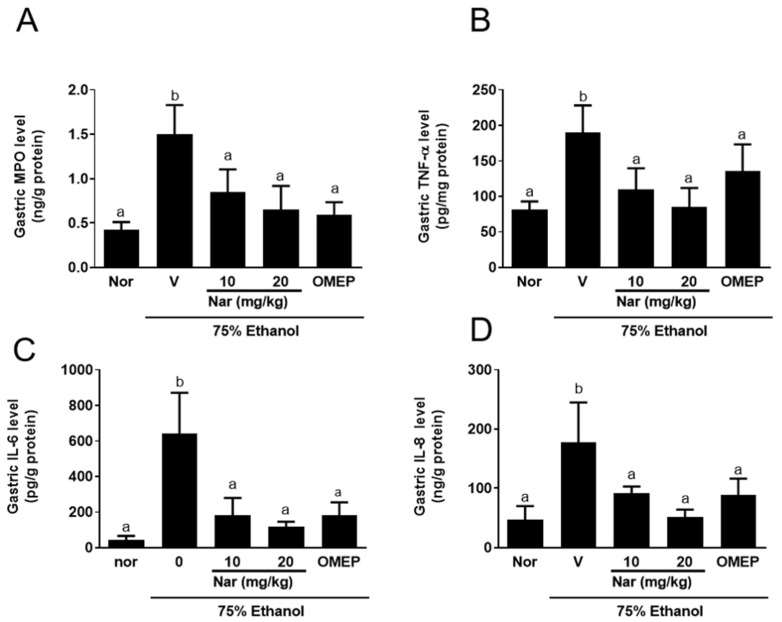

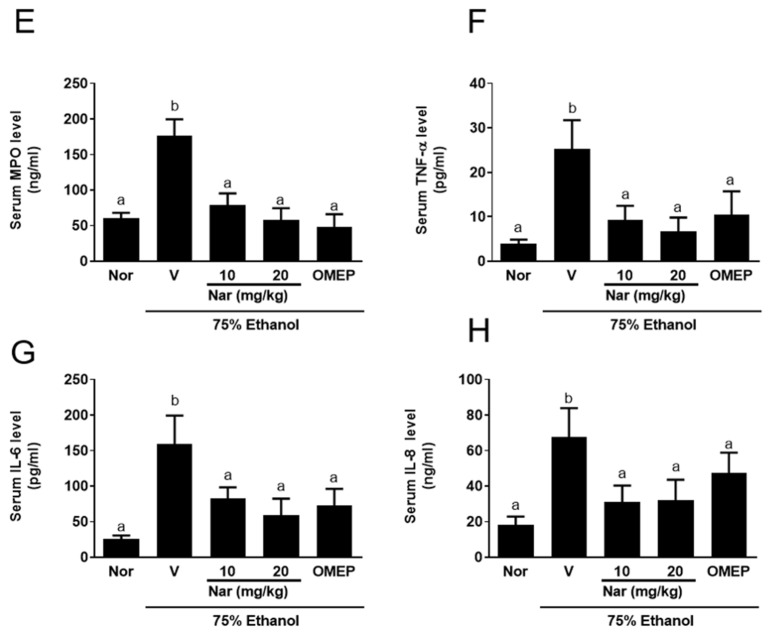
Effect of naringenin on myeloperoxidase (MPO) activity and cytokine levels (TNF-α, IL-6, and IL-8) in the stomach homogenate (**A**–**D**) and in serum (**E**–**H**). The vehicle (10% dimethyl sulfoxide (DMSO) and 90% glyceryl trioctanoate), naringenin (Nar, 10 and 20 mg/kg) and omeprazole (OMEP, 20 mg/kg; used as a positive control) were given orally for a period of 3 days. After fasting for 12 h prior to the experiment, mice were fed orally with ethanol (0.5 mL/100 g body weight) to induce the acute ulcer. After 4 h, mice were sacrificed. The data obtained from individual animal samples per group were averaged (*n* = 6); values represent mean ± standard deviation (SD). Statistical comparison was analyzed by a one-way ANOVA followed by Tukey’s multiple comparison tests. Bars not sharing a common letter represent a statistically significant difference from each other (*p* < 0.05). In the normal control group, the mice only received vehicle (Normal). Nor, normal; V, vehicle.

We investigated the activity of myeloperoxidase (MPO), a biochemical marker of neutrophil infiltration, after ethanol induction. As shown in Figure 5A,E, the MPO activity in the stomach and serum was significantly increased after ethanol administration, which confirmed that ethanol causes the activation and infiltration of neutrophils. However, pretreatment naringenin significantly attenuated MPO activity in gastric homogenates (Figure 5A) and serum (Figure 5E). Mice that were treated with omeprazole also had a significant reduction of the MPO activity compared to the control group. To examine this further, we measured the levels of important inflammatory factors e.g., tumor necrosis factor-α (TNF-α), interleukin-6 (IL-6), and interleukin-8 (IL-8) in gastric homogenates (Figure 5B–D) and serum (Figure 5F–H) to evaluate anti-inflammatory activity of naringenin. The levels of these cytokines in gastric tissue and serum were dramatically increased in mice with acute gastric injury induced by ethanol. Intriguingly, pretreatment with naringenin effectively downregulated TNF-α, IL-6, and IL-8 in gastric tissue and serum. These observations indicate that naringenin exerts gastro-protective physiological activities which aid in the alleviation of neutrophil infiltration and inflammation.

### 2.5. Effect of Naringenin on the Mitogen-Activated Protein Kinases (MAPKs) and Nuclear Factor-κB (NF-κB) Pathways in KATO III Cells Activated by Ethanol

**Figure 6:**
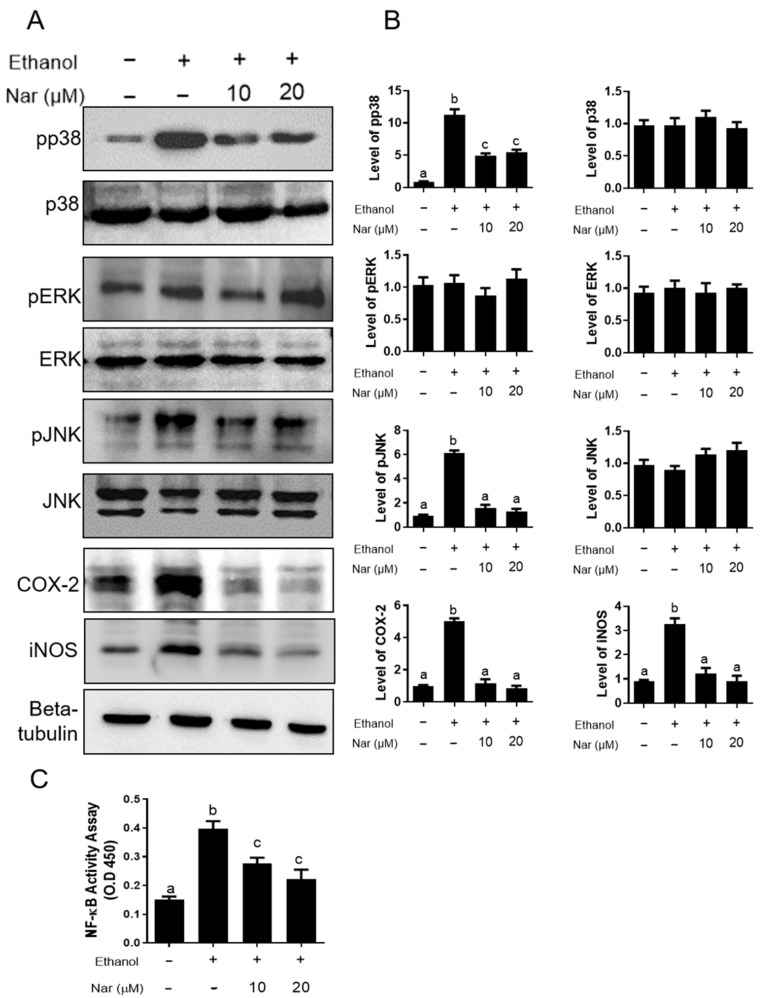
Phosphorylation of MAPKs and translocation of NF-κB p65 in ethanol-stimulated KATO III. (**A**) Levels of phosphorylated p38 MAPK, ERK, JNK, COX-2 and iNOS from ethanol-stimulated KATO III cellular lysates in the absence or presence of naringenin (Nar, 10 and 20 μM). Representative images from three independent experiments with similar results. β-tubulin was used as a loading control. (**B**) The bar graph at the quantitation of western blot signal intensities are presented as the mean ± SEM of 3 wells from a representative experiment. The levels of each target protein were normalized to β-tubulin loading control and presented as the relative expression levels to the no treatment control group (**C**) NF-κB p65 DNA-binding activity in nuclear extracts of KATO III was determined by using the TransAM kit, representing the optical density at 450 nm (OD450) values which were depicted as means ± SD from triplicate samples for each treatment. Statistical comparison was analyzed by a one-way ANOVA, followed by Tukey’s multiple comparison tests. Bars not sharing a common letter represent a statistically significant difference from each other (*p* < 0.05).

**Figure 7:**
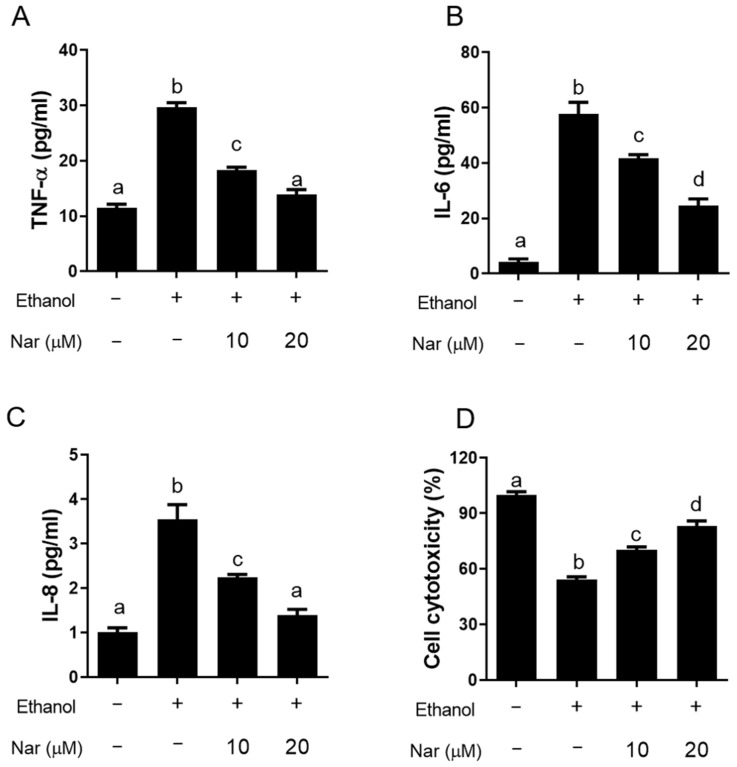
Effects of naringenin on the expression of inflammatory cytokine expression in ethanol-stimulated KATO III cells. Cells (0.5 × 10^6^) were incubated with ethanol in the absence or presence of naringenin (Nar, 10 and 20 μM). (**A**–**C**) Cytokine concentrations (TNF-α, IL-6, and IL-8) in the medium were assessed by ELISA, respectively. (**D**) Effects on the cytotoxicity. Cell viability was determined by MTT assay. All data were shown as mean ± standard deviation (SD) (*n* = 3). Statistical comparison was analyzed by a one-way ANOVA followed by Tukey’s multiple comparison tests. Bars not sharing a common letter represent a statistically significant difference from each other (*p* < 0.05).

To explore the gastroprotective mechanism of naringenin on against ethanol-induced GU, we further investigated the effect of naringenin on the activation of MAPK and NF-κB in KATO III cells stimulated by ethanol. The phosphorylation levels of p38 MAPK, extracellular signal-related kinase (ERK), and c-Jun N-terminal kinase (JNK) were analyzed by Western blot. Additionally, the nuclear translocation of NF-κB was quantified using a p65 binding assay. As shown in Figure 6A, naringenin significantly decreased the phosphorylation of p38 and JNK when cells were induced by ethanol, while the expression of pERK appeared to remain unchanged regardless of treatment. The activation of NF-κB is an important transcription factor within the inflammatory response. NF-κB plays a key role in regulating the expression of cyclooxygenase-2 (COX-2), inducible nitric oxide synthase (iNOS), and numerous inflammatory cytokines [30]. To investigate the effects of naringenin on NF-κB activity, an NF-κB binding assay was used. Elevated levels were detected upon ethanol stimulation, however, naringenin treatment significantly reduced NF-κB activity (Figure 6C). In addition, exposure to ethanol showed a significant increase in COX-2 and iNOS in KATO III cells (Figure 6B). Pretreatment with naringenin significantly decreased the expressions of COX-2 and iNOS in a dose-dependent manner. To investigate whether naringenin could affect ethanol-induced gastritis, we examined the effects on the secretion of TNF-α, IL-6, and IL-8 expression in ethanol-stimulated KATO III cells (Figure 7A–C). Ethanol treatment induced a significant increase in the secretion of TNF-α, IL-6, and IL-8 expression in KATO III cells. Additionally, pretreatment with naringenin was able to significantly decrease ethanol-induced expression of these cytokines. It was also determined that naringenin can reduce cytotoxicity after exposure to ethanol (Figure 7D). These observations indicate that naringenin suppresses the ethanol-induced activation of nuclear factor-κB (NF-κB), pp38 MAPK, and JNK to regulate inflammatory mediators, such as iNOS, COX-2, TNF-α, IL-6, and IL-8. These results indicate that naringenin may inhibit inflammation through the downregulation of inflammatory mediators, including iNOS, COX-2, TNF-α, IL-6, and IL-8, by suppressing NF-κB/MAPK-related signaling pathways.

## 3. Discussion

The present study investigated the gastroprotective effects of naringenin against ethanol-induced GU in mice. In this study, a single oral administration of 75% ethanol was found to cause acute gastric bleeding and hemorrhagic lesions in mouse stomach, confirming the detrimental effects of ethanol on gastric mucosa. We found that naringenin-pretreated animals showed less macroscopic damage, possessed a lower gastric injury index (hemorrhagic damage, epithelial cell loss, and edema with leucocytes), decreased oxidative stress, reduced pro-inflammation, and led to a higher pH value and mucus content compared with mice that received ethanol alone. Therefore, the oral administration of naringenin effectively protected against ethanol-induced acute gastric mucosal injury in mice.

Lüllmann et al. [31] pointed out that increased hydrogen ion concentration is an aggressive factor that facilitates gastric injury. The study showed that the gastric pH value of ethanol-treated mice was significantly reduced compared with mice that received ethanol alone. However, naringenin pretreatment resulted in an increase in the gastric pH compared to mice that received ethanol alone. Naringenin is part of the flavonoid family. Flavonoids have previously been shown to play an important role in gastroprotection by raising the pH value of the gastric juice [32,33].

Naringenin has a free-radical-scavenging ability, which can prevent free radicals from attacking amino acids [34]. The polarity of naringenin can promote its adhesion to the lipid bilayer, thereby reducing the formation of free radicals and protecting cell membranes [35]. Oxidative stress is involved in the pathogenesis of ethanol-induced gastric mucosal damage, which is caused by the imbalance between ROS and antioxidants [36]. Ethanol causes severe oxidative stress in gastric tissues, which is manifested by an increasing MDA content and decreasing gastric GSH content [37]. Consistent with previous results, ethanol significantly increased MDA content and decreased GSH content in the gastric tissues and serum in the study. Furthermore, the level of the antioxidant enzyme, SOD, was clearly reduced in the ethanol-treated group compared to the normal control group. Here, we observed that pretreatment with naringenin significantly inhibited the increase in lipid peroxidation, slightly increased the mucosal GSH content, and slightly enhanced the activity of SOD in the gastric tissues and serum. These results suggested that naringenin can act as an antioxidant to further reduce ethanol-induced gastric injury. Previous studies have also shown that naringenin inhibited microsomal lipid peroxidation [38], nonenzymatic lipid peroxidation [39], and ascorbic-acid-induced MDA formation [40]. Van Acker et al. [41] pointed out that naringenin can restore glutathione-dependent protective effects and prevent lipid peroxidation in α–tocopherol-deficient liver microsomes. In addition, previous studies have shown that naringenin supplementation significantly decreased the levels of MDA in rats with ethanol-induced hepatotoxicity [34]. NO is produced by nitric oxide synthases (NOS), which includes the constitutive nitric oxide synthase (cNOS) and inducible nitric oxide synthase (iNOS). cNOS can continuously release a low level of NO under physiological conditions [42], while iNOS can produce a high level of inducible NO after being activated by inflammatory cytokines that leads to vascular microcirculation disturbance and gastric mucosal damage [43,44,45]. In the present study, ethanol significantly increased NO levels in the gastric tissue and serum when compared to the normal group. However, pretreatment with naringenin prominently decreased NO levels, suggesting that naringenin reduces vascular microcirculation disturbance and gastric mucosal damage by reducing NO levels, and thus has a gastroprotective effect. This finding is consistent with the previous observation that naringenin chalcone attenuated lipopolysaccharide (LPS)-induced increase in NO, TNF-α, and monocyte chemoattractant protein-1 (MCP-1) production in RAW 264 macrophages [46].

MPO is mainly released by neutrophils. Therefore, it is a biomarker of neutrophil-dependent inflammation [47,48]. Previous studies showed ethanol causes an increase in mucosal MPO activity, which indicates an increase in neutrophils that secrete ROS [49,50]. In the present study, ethanol-induced mice showed an increase in MPO activity compared with the normal control. Pretreatment with naringenin before ethanol induction suppressed the release of MPO compared to the ulcer control group, who received the vehicle alone. Therefore, the degree of inflammation induced by neutrophils was also inhibited. These findings are consistent with the previous observations that naringenin has been shown to inhibit MPO activity [51].

In addition, ethanol can cause inflammation and release a high number of inflammatory cytokines [52]. Excessive intake of ethanol causes inflammation, and the numbers of macrophages and lymphocytes increase at the site of inflammation [53]. Therefore, ethanol intake increases the level of serum IL-8 and stimulates the gastric epithelium to cause inflammation by inducing proinflammatory cytokines (e.g., TNF-α and IL-6). This leads to a high number of infiltrating immune cells, especially neutrophils, which ultimately leads to gastritis [54,55]. Consistent with previous results, our results indicate that administration of ethanol significantly elevated the levels of MPO, TNF-α, IL-6, and IL-8 in gastric tissues and serum. This study has shown that pretreatment with naringenin distinctly altered the levels of proinflammatory cytokines that are increased by ethanol induction in gastric tissue and serum. This indicates that the therapeutic effect of naringenin on ethanol-induced GU is involved in the inhibition of local inflammatory processes. This finding is consistent with the previous observation that naringenin has anti-inflammatory effects [56,57,58,59,60]. Pinho-Ribeiro et al. [56] pointed out that naringenin attenuates carrageenan-induced oxidative stress, hyperalgesic cytokines (IL-33, TNF-α, and IL-1β), and NF-κB activation in the paw skin. Azuma et al. [57] showed that naringenin has anti-inflammatory effects in the dextran sulfate sodium (DSS)-induced colitis. Dou et al. [58] also demonstrated naringenin can improve DSS-induced murine colitis through inhibiting toll-like receptor 4 (TLR4) protein and NF-κB activity, downregulating the expression of inflammatory mediators (iNOS, ICAM-1, MCP-1, COX-2, TNF-α, and IL-6) and inhibiting the production of inflammatory cytokines (TNF-α and IL-6). Shi et al. [59] showed that naringenin attenuated the allergen-induced murine airway inflammation through NF-κB inhibition. Lastly, Hämäläinen et al. [60] suggested that naringenin reduced LPS-induced nuclear translocation of NF-κB and iNOS expression in RAW 264 macrophages.

To further investigate the mechanism of naringenin against ethanol stress, we first targeted NF-κB activity in KATO III cells. In this study, we found ethanol remarkably elevated NF-κB activity, which was significantly suppressed by pretreatment of naringenin. In addition, ethanol-stimulated the expression of inflammatory cytokines TNF-α, IL-6, and IL-8, and the proteins of COX-2 and iNOS in stomach epithelial KATO III cells. However, naringenin downregulated these cytokines and proteins in a dose-dependent manner. Therefore, we speculate that the mechanism of action of naringenin is to deactivate p38 MAPK, pJNK, and NF-κB to maintain the cellular homeostasis under ethanol stress on KATO III cells. Zhang et al. [61] showed that naringenin exerts a pharmacological effect against the progression of cardiac hypertrophy induced by pressure overload through inhibiting JNK, ERK, and PI3K/Akt signaling pathways.

## 4. Material and Methods

### 4.1. Ethanol-Induced Gastric Injury

Male Balb/c mice (6–7 weeks old; 20–25 g) were purchased from National Laboratory Animal Center (Taipei, Taiwan) and used after one week of quarantine and acclimatization. The animals were housed under environmentally controlled conditions (22 ± 2 °C; relative humidity, 50 ± 5%) with a 12 h light/dark cycle under specific pathogen-free (SPF) conditions. The mice were provided with standard rodent chow and sterilized tap water ad libitum. The experimental procedures were conducted in compliance with the Institutional Animal Care and Use Committee (IACUC) of National Chung Hsing University (IACUC-109039). Mice were randomly divided into five groups (*n* = 6 in each group). The (i) normal and (ii) ulcer control groups received vehicle (10% dimethyl sulfoxide (DMSO) and 90% glyceryl trioctanoate) throughout the course of the experiments. The prevention groups intragastrically received different doses of (iii, iv) naringenin (10 and 20 mg/kg, dissolved in 10% DMSO and 90% glyceryl trioctanoate) and (v) omeprazole (20 mg/kg, reference drug, dissolved in 10% DMSO and 90% glyceryl trioctanoate), respectively, for a period of 3 days. After fasting for 12 h before the experiment, (ii) ulcer mice group and (iii, iv, v) prevention mice groups were fed orally with 75% ethanol (0.5 mL/100 g body weight) to induce the acute ulcer, while the (i) normal group received water only. Four hours after induction of ulcers, the mice were anesthetized, and their blood samples were collected through cardiac puncture under isoflurane anesthesia. Then, the stomachs were rapidly removed, opened along the greater curvature, and rinsed with ice-cold saline to remove the gastric contents and blood clots to make macroscopic observations on the extent of gastric damage. After that, each stomach was divided into two parts, one part of the stomach was immersed in 10% formaldehyde for histological evaluation, and the other part of the stomach tissue was stored at −80 °C for biochemical analysis. The blood samples were then centrifuged for 10 min at 2500× *g* to obtain clean serum, which was stored at −20 °C for biochemical analysis.

### 4.2. Macroscopic Observation

Stomachs were removed and cut the larger curvature, rinsed thoroughly in saline solution, and macroscopically imaged. Macroscopic damage was assessed by blinded observers, and scored as follows [62]: 0, no lesions; 1–2, small lesions; 3–4, small ulcer; 5–6, large ulcer; 7, full of ulcers.

### 4.3. Histological Analysis

Stomach tissues were excised and fixed in a 10% (*v/v*) formalin solution and then paraffin-embedded, as previously described [63]. Paraffin-embedded sections at 5 μm thickness were stained with hematoxylin and eosin (H&E) for histological evaluation. Then, the pathological changes in gastric tissues were observed under a microscope (CKX41; Olympus, Tokyo, Japan). The hemorrhagic damage, epithelial cell loss and edema with leucocytes of gastric mucosa were scored, and the degrees of lesions were graded from one to five depending on severity: 1 = minimal (<1%); 2 = slight (1–25%); 3 = moderate (26–50%); 4 = moderate/severe (51–75%); 5 = severe/high (76–100%).

### 4.4. Determination of Gastric pH in Gastric Juice

The stomach contents were collected after ligating the pylorus and centrifugation at 3000× *g* rpm for 10 min at 15 °C. The pH values of gastric juice acid were analyzed [64].

### 4.5. Gastric Wall Mucus Determination

The procedure described by Corne et al. [65] was used to quantify mucus on the gastric wall. The glandular segments of each stomach were weighed and immediately added to 1% Alcian blue solution (sucrose solution buffered with sodium acetate, pH 5). Excess dye was removed with a sucrose solution. The dye, which adhered to mucus on gastric wall, was extracted with a 4 mL solution of magnesium chloride mixed with an equal volume of diethyl ether. The resulting emulsion was centrifuged, and the absorbance was measured using a spectrophotometer at a wavelength of 580 nm. The quantity of Alcian blue extracted per gram of glandular tissue was calculated. The quantity of Alcian blue was determined by linear regression analysis and a calibration curve that was generated with different concentrations of Alcian blue. The results were expressed as micrograms of Alcian blue extracted per gram of gastric tissue.

### 4.6. Determination of NO Level

Total protein was isolated from 100 mg of gastric tissue by homogenization in 1 mL of a tissue protein extraction reagent (Pierce, Rockford, IL, USA) with protease inhibitors (Roche, Indianapolis, IN, USA). The supernatants obtained after the centrifugation of homogenates (10,000× *g* for 5 min at 4 °C) were used for protein assays, with the remainder of the supernatant stored at −80 °C for further analysis. The protein concentrations were quantified by a bicinchoninic acid protein assay kit (Thermo Fisher Scientific, Waltham, MA, USA). The level of NO in gastric tissue and serum of the experimental mice were evaluated as total nitrate/nitrite using Griess reagent [66] and the operational processes were measured following the NO kit instructions. In brief, 50 μL of tissue supernatant and serum was added to 50 μL Griess reagent [0.1% *N*-(1-naphthyl) ethylenediamine dihydrochloride, 1% sulphanilamide and 2.5% H_3_PO_4_] and mixed. After incubation at room temperature for 10 min, the absorbance was measured at 540 nm on a microplate reader (TECAN, Durham, NC, USA).

### 4.7. Determination of MDA, GSH, SOD, and Myeloperoxidase (MPO) Activities

The concentrations of MDA, GSH, SOD, and MPO in supernatants of stomach tissue homogenate or serum were determined using commercial kits purchased from the MyBiosource Company (MDA: MBS741034, MyBioSource, San Diego, CA, USA; GSH: MBS700004, MyBioSource, San Diego, CA, USA; SOD: MBS265351, MyBioSource, San Diego, CA, USA; MPO: MBS700747, MyBioSource, San Diego, CA, USA). All procedures were performed according to the manufacturer’s recommendations.

### 4.8. Cytokine Measurements

The concentrations of TNF-α, IL-6, and IL-8 in supernatant of stomach tissue homogenate or serum were determined using an enzyme-linked immunosorbent assay (ELISA) according to the manufacturer’s recommendations (TNF-α: Cat. No. 430904, Biolegend, San Diego, CA, USA; IL-6: Cat. No. 431304, Biolegend, San Diego, CA, USA; IL-8: Cat. No. MBS1601073, MyBiosource Company, San Diego, CA, USA).

### 4.9. Cell Culture and Cell Viability Assay

KATO III cells provided by the American Type Culture Collection (ATCC) were cultured in RPMI 1640 (Sigma) containing 1% antibiotic-antimycotic and 10% fetal bovine serum at 37 °C. For the assay of cytotoxicity, KATO III cells were pretreated with naringenin (10 or 20 μM) for 2 h prior to being treated with ethanol (50 μL/mL) for an additional 24 h. Cells treated with 0.1% of DMSO were a vehicle control. Cell viability was assayed using Cell Counting Kit-8 (CCK-8) assay kit according to the manufacturer’s protocol.

### 4.10. Analysis of Cytokine Secretion In Vitro

To measure anti-inflammatory effect of naringenin, KATO III cells were pretreated with naringenin (10 or 20 μM) for 2 h prior to being treated with ethanol (50 μL/mL) for an additional 24 h. Cells treated with 0.1% of DMSO were a vehicle control. Cytokine concentrations of TNF-α, IL-6, and IL-8 in the culture medium were analyzed by ELISA kits.

### 4.11. Western Blotting

KATO III cells were pretreated with naringenin (10 or 20 μM) for 2 h prior to being treated with ethanol (50 μL/mL). Then, cell suspension was collected at 1 h after treatment of ethanol to determine the phosphorylation of MAPK pathways, and at 18 h after treatment of ethanol for COX-2 and iNOS measurement. RIPA cell lysis buffer containing protease inhibitor was used to lyse cells on ice for 30 min to obtain the cell lysate, followed by bicinchoninic acid (BCA) assay for determination of the protein concentration. 40 μg of protein lysate was boiled and loaded into 8–10% gradient SDS-PAGE gels, and then electro-transferred to nitrocellulose membranes. Membranes were hybridized with primary antibodies that recognize p-p38 (clone 3D7, 1:1000; Cell Signaling Technology, Beverly, MA, USA), p38 (clone D13EE1, 1:1000; Cell Signaling Technology, Beverly, MA, USA), p-ERK (clone E-4, 1:1000; Santa Cruz Biotechnology, CA, USA), ERK (clone H-72, 1:1000; Santa Cruz Biotechnology, CA, USA), p-JNK (clone 81E11, 1:1000; Cell Signaling Technology, Beverly, MA, USA), and JNK (Cat# 9252, 1:1000; Cell Signaling Technology, Beverly, MA, USA), COX-2 (clone SP21, 1:1000; Thermo Fisher Scientific, Waltham, MA, USA), iNOS (PA3-030A, 1:2000; Thermo Fisher Scientific, Waltham, MA, USA), and β-tubulin (Cat #PA5-16863, 1:1000; Thermo Fisher Scientific, Waltham, MA, USA) at 4 °C overnight. The horseradish peroxidase (HRP)-conjugated corresponding secondary antibody (1:10,000; Jackson ImmunoResearch Laboratories, West Grove, PA, USA) at 4 °C, incubating for an additional night prior to developing with ECL reagent (GE Healthcare Life Sciences, Piscataway, NJ, USA) and visualizing with the Hansor Luminescence Image System (Taichung, Taiwan). The band density was measured with the ImageJ v1.47 program for Windows from the National Institute of Health (NIH) (Bethesda, Rockville, MD, USA).

### 4.12. Preparation of Nuclear Extracts and Measurement of NF-κB Activity

KATO III cells were pretreated with naringenin (10 or 20 μM) for 2 h prior to being treated with ethanol (50 μL/mL). Cell lysates were collected at 60 min after treatment of ethanol to determine the NF-kb activity. The nuclear extracts were prepared using the NE-PER Nuclear and Cytoplasmic Extraction system (Thermo Fisher Scientific, Waltham, MA, USA). For each assay, a total of 10 μg of nuclear extract was used in a TransAM NF-κB p65 ELISA kit (Active Motif, Carlsbad, CA, USA). The procedure was performed according to the manufacturer’s instructions.

### 4.13. Statistical Analysis

Data were expressed as mean ± SEM. One-way ANOVA with a post hoc Tukey’s test was used to compare multiple experimental groups with GraphPad Prism v5.0 software (GraphPad Software, Inc., San Diego, CA, USA). A *p*-value of less than 0.05 was considered a significant difference.

## 5. Conclusions

**Figure 8:**
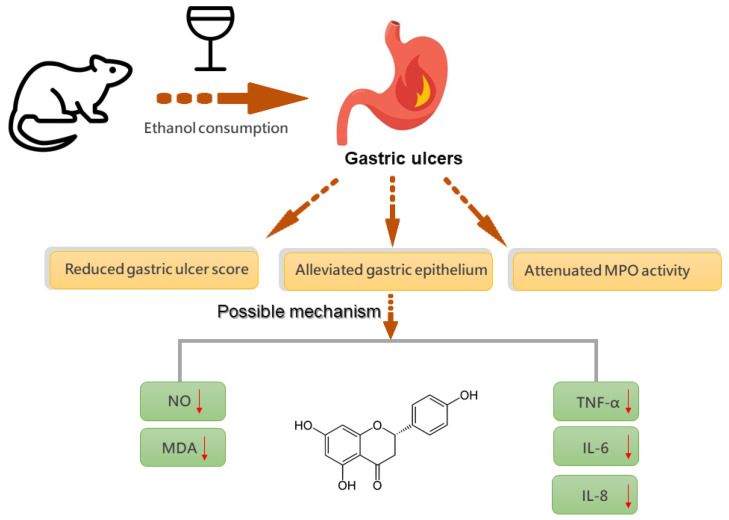
The gastroprotective mechanism of naringenin against ethanol-induced gastric ulcers.

The present study focused on the protective effects of naringenin in gastric mucosa. Ethanol was used to induce gastric mucosal injury and the effect of naringenin was investigated. The present study showed that naringenin exerted significant protective effects against gastric mucosal damage, as seen by the significant reduction in oxidative stress and pro-inflammatory cytokines induced by ethanol (Figure 8). Therefore, we suggest that naringenin might be a useful candidate in the clinical treatment of gastric disorders.

## Data Availability

The data that support the findings of this study are available from the corresponding author upon reasonable request.
